# An Analytical Model of IaaS Architecture for Determining Resource Utilization †

**DOI:** 10.3390/s24092758

**Published:** 2024-04-26

**Authors:** Slawomir Hanczewski, Maciej Stasiak, Michal Weissenberg

**Affiliations:** Faculty of Computing and Telecommunications, Poznan University of Technology, 60-965 Poznan, Poland; maciej.stasiak@put.poznan.pl (M.S.); michal.weissenberg@put.poznan.pl (M.W.)

**Keywords:** cloud computing, IaaS, analytical model

## Abstract

Cloud computing has become a major component of the modern IT ecosystem. A key contributor to this has been the development of Infrastructure as a Service (IaaS) architecture, in which users’ virtual machines (VMs) are run on the service provider’s physical infrastructure, making it possible to become independent of the need to purchase one’s own physical machines (PMs). One of the main aspects to consider when designing such systems is achieving the optimal utilization of individual resources, such as processor, RAM, disk, and available bandwidth. In response to these challenges, the authors developed an analytical model (the ARU method) to determine the average utilization levels of the aforementioned resources. The effectiveness of the proposed analytical model was evaluated by comparing the results obtained by utilizing the model with those obtained by conducting a digital simulation of the operation of a cloud system according to the IaaS paradigm. The results show the effectiveness of the model regardless of the structure of the emerging requests, the variability of the capacity of individual resources, and the number of physical machines in the system. This translates into the applicability of the model in the design process of cloud systems.

## 1. Introduction

The telecommunications market, in its constant state of evolution, demonstrates a relentless drive to meet increasingly demanding user needs. This evolution is clearly visible in the field of wireless access networks, where the advent of 4G and 5G technologies has ushered in a new era of seamless voice transmission and lightning-fast data transfer. Declining device and data costs have greatly expanded the network services sector. Today, IP traffic is a major contributor to the flow of data on the Internet, demonstrating the ubiquitous role of wireless access networks in modern communications ecosystems [1]. Nevertheless, it is important to realize that wireless access networks are only one aspect of a broader system dedicated to serving user requirements. High-performance backbone networks and high-performance servers form the backbone of this infrastructure, facilitating seamless service delivery processes. Servers, often consolidated in sprawling data centers, play a key role in streamlining service management operations. Leading content-delivery networks boast extensive sets of servers, comprising thousands of units, enabling them to offer a diverse set of services to end users. The advent of cloud computing [2,3,4,5] has further revolutionized service availability and deployment, with cloud-based resources now available as on-demand instances. This allows a myriad of content and applications to be made available quickly and efficiently. As a result, cloud-based services have penetrated both enterprise-class service providers and individual users seeking customized server resources or storage capacity for personal data management. Other examples of data-processing approaches include Edge Computing [6,7,8] and Fog Computing [9,10,11,12], which are extensively described in the literature. Fog and Edge Computing approaches are widely used, especially in real-time data processing, where minimizing latency and providing excellent scalability are becoming increasingly important. Fog Computing, thanks to its distributed infrastructure, allows it to complement Edge Computing and extends its capabilities by providing a computing infrastructure layer between edge devices and the cloud. Consequently, it provides additional computing resources and services to edge devices. At the same time, with huge requirements for computing power or a service where the user requires access to infrastructure in the form of virtual machines, cloud solutions remain irreplaceable. At the same time, cloud solutions remain indispensable in handling a huge demand for computing power or services, where the user requires access to infrastructure in the form of virtual machines.

Cloud computing, epitomized by the on-demand resource-delivery model, is an indispensable part of users’ lives. Characterized by the ability to rapidly scale computing resources up or down in response to user demand, it offers unparalleled flexibility and cost efficiency. Prominent among the various cloud service models is Infrastructure as a Service (IaaS), which provides users with unlimited access to virtualized computing resources such as virtual machines, storage, and network components via the Internet. With IaaS, users retain full autonomy to provision and manage resources, while not having to invest in their own physical infrastructure.

The National Institute of Standards and Technology (NIST) has delineated the essential attributes of IaaS, defining it as “the capability provided to the consumer to provision processing, storage, networks, and other fundamental computing resources where the consumer is able to deploy and run arbitrary software, which can include operating systems and applications. The consumer does not manage or control the underlying cloud infrastructure but has control over operating systems, storage, deployed applications, and possibly select networking components” [2].

However, the effectiveness of cloud computing systems depends on judicious resource allocation and vigilant monitoring. Overloading individual resources can result in degraded performance of the entire system and increased failure rates. Thus, an optimal use of resources is sought, according to the assumptions made while avoiding their maximum use in longer time sequences. For example, constant CPU overload can mean system bottlenecks or software inefficiencies. Similarly, disk space allocation requires a high degree of caution, and experts recommend a buffer of 10–15% of free disk space to prevent system instability. The effective management of cloud computing infrastructure involves dealing with a myriad of challenges, chief among which are resource availability and energy efficiency. Service providers have to deal with the complexity of scaling physical server resources to meet growing user demands, especially in IaaS offerings. Moreover, optimizing energy consumption and managing heat dissipation while servers are running remains a pressing issue. Data centers, the backbone of cloud infrastructures, are significant consumers of energy, prompting ongoing sustainability and energy efficiency efforts. According to the International Energy Agency (IEA), the entirety of data centers worldwide consumed about 200 TWh of electricity in 2020 [13]. To this end, advanced load-balancing algorithms, including Opportunistic Load Balancing (OLB), Round Robin (RR), and Central Load Balancing Decision Models (CLBDM), have been developed to ensure equitable use of resources and minimize energy losses [14,15,16,17]. A detailed description of the various load-balancing models is provided in Section 2.1.

To address the identified challenges, the paper presents a general approach to predicting resource utilization in the cloud. The authors developed a model (the ARU method) to predict the utilization of basic cloud system resources such as CPU cores, disk storage, RAM, and available bandwidth when observing the system.

### 1.1. Related Works

The topic of resource management and resource usage prediction has been widely addressed in the literature on the subject. In the paper [18], the authors proposed an intelligent Regressive Ensemble Approach for Prediction (REAP). The solution integrates feature selection and resource usage prediction techniques to achieve high performance. The authors verified the accuracy of their solution in a real cloud environment. The main parameter analyzed was CPU utilization, and the model was characterized by very high accuracy and speed. A method proposed by [19] focuses on load prediction for energy-efficient consolidation of virtual machines in cloud data centers. The authors introduce LiRCUP, a technique based on linear regression for predicting CPU utilization on each host. LiRCUP also facilitates the prediction of underloaded hosts and the migration of VMs to other hosts. Similarly, Ref. [20] presents a model for predicting resource instances in real-time cloud environments. They classify workloads based on trend degree (TD) and utilize a hidden Markov model (HMM) to forecast cloud resource usage using historical and current data. In contrast, Ref. [21] proposes an ensemble prediction algorithm to forecast energy efficiency in cloud environments. Their model operates at various levels, employing prediction models such as moving average, linear regression, exponential smoothing, and double exponential smoothing. Another distinctive approach is illustrated by [22], where the authors develop a prediction-based resource provisioning technique using neural networks (NNs) and linear regression (LR) specifically for the Amazon EC2 cloud. Article [23] attempts to predict real-time resource utilization for IaaS-based cloud systems using the ARIMA method for requests following a Gaussian distribution. Their model selection is based on minimum Akaike Information Criterion (AIC) values, with evaluation performed on the FastStorage dataset. In [24], an ensemble model for load prediction is presented, demonstrating improved accuracy and root mean square error (RMSE) compared to baseline studies. The ensemble, named the “Ensemble based workload prediction mechanism”, employs stack generalization and base classifiers such as k-nearest neighbors (KNN) and decision trees, showing promising performance enhancements. In the article [25], the authors presented an approach to predicting resource utilization in the cloud at the level of individual tasks and resources. The proposed solution uses methods from the field of machine learning to create predictive models based on historical data. The authors used real-world datasets in their study and, based on these datasets, showed that the approach they developed improves the prediction accuracy of the duration of emerging requests compared to a simple linear regression approach. Based on their evaluation, it was shown that in a typical case, a 20% reduction in prediction error is possible and that improvements above 89% are among the best cases. For the median case, the model predicted the duration of tasks in the cloud with an error factor of 0.80 (i.e., 20% less prediction error). The best 5% of cases achieved an error rate of 0.11 (i.e., 89% lower prediction error).

In summary, a number of approaches to managing and predicting resource usage in cloud systems can be found in the literature. Most of the proposed solutions are based on historical data and its analysis for prediction using artificial intelligence algorithms. The approach proposed by the authors differs and is based on the intensity of incoming requests, request characteristics, and physical machine parameters, followed by analytical modeling using Markov processes.

### 1.2. Research Contribution

The main achievements of this article are as follows:

- An analytical model in the form of a method, called ARU method, is developed to determine the average use of each cloud system resource (RAM (R), disk (D), processor (P), bandwidth (B)) during its operation.
- In order to develop the proposed algorithm, models of multi-service systems were used: a model of multi-service resources with full availability, a model of multi-service resources with limited availability, and the methodology of fixed points.
- A simulation model of a cloud system based is developed on requests for four parameters (R, D, P, B) in order to obtain information on the use of individual resources of physical machines and indicate the impact of individual resources on the rejection of requests;
- The results obtained using the model are compared with the results obtained using the simulator developed by the authors.

## 2. Cloud Computing Structure

**Figure 1:**
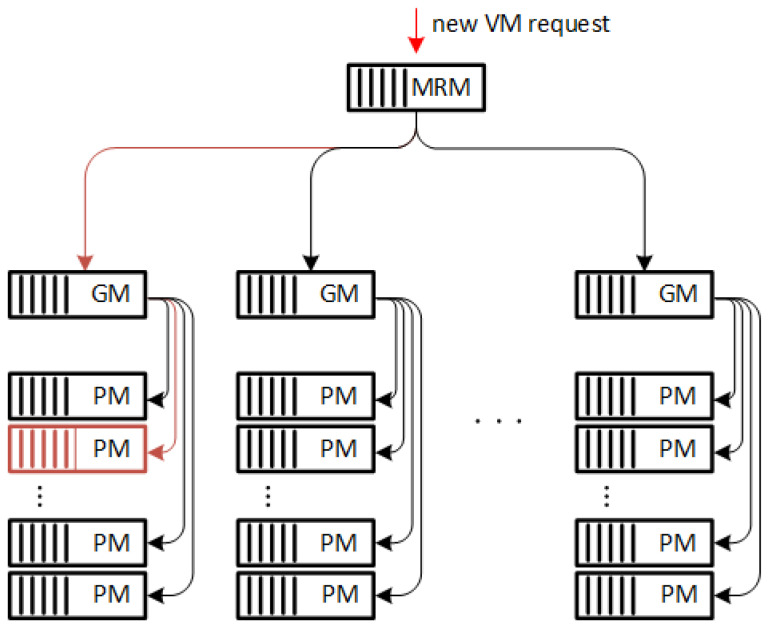
The process of creating a virtual machine in a cloud system.

The concept of creating a virtual machine in a cloud environment is described by the author in the article [26]. The whole concept is depicted in the schematic drawing Figure 1. This diagram consists of two main components: the management part (consisting of the Main Resource Manager (MRM) and the Group Manager (GM)) and the Physical Machine (PM) itself, represented as a server [27].

### 2.1. Management in the Cloud

In a cloud computing environment, the Main Resources Manager (MRM) and Group Manager (GM) are responsible for managing the allocation and utilization of resources within the cloud. The introduction of such a division is intended to simplify the management of resources by dividing them into individual groups.

The MRM is responsible for managing the physical resources of the cloud, such as servers, storage, and networking equipment. It ensures that these resources are available and accessible to users in the cloud. The MRM also monitors the utilization of these resources and makes decisions on resource allocation based on user demand.

The GM, on the other hand, manages the virtual resources within the cloud. This includes virtual machines, applications, and other services. The GM ensures that these resources are provisioned and available to users as needed and also monitors their utilization and performance.

Both the MRM and GM work together to ensure efficient and effective utilization of resources within the cloud environment. They use various algorithms and strategies to manage resource allocation and utilization, such as load balancing and auto-scaling, to optimize performance and minimize downtime.

There are several algorithms used in cloud computing to distribute Virtual Machines (VMs) to Physical Machines (PMs). Some of the commonly used algorithms are [14,15,16,17]:

- Round-robin: This algorithm distributes VMs in a round-robin fashion across the available PMs. It ensures an even distribution of VMs across the PMs and prevents the overloading of any single PM.
- Opportunistic load balancing (OLB): This algorithm dynamically monitors the load on each PM and migrates VMs from overloaded PMs to underloaded ones to balance the load. It makes use of statistical models to predict the future load on PMs.
- Central load balancing decision model (CLBDM): This algorithm uses a central controller to balance the load across the PMs. The controller has access to the load information of all PMs and makes decisions on where to place VMs based on the current and predicted future load.
- Ant Colony Optimization (ACO): This algorithm is inspired by the behavior of ants in finding the shortest path between two points. In the cloud computing environment, ACO can be used to find the optimal placement of VMs based on resource utilization, energy consumption, and other criteria.
- Genetic algorithm (GA): This algorithm uses a population-based approach to find the optimal solution for VM placement. It starts with an initial population of VM placement solutions and evolves them using mutation, crossover, and selection operations to find the fittest solution.
- Uniform distribution: This is an even distribution that tends to average each device’s resource usage. This approach was taken into account by the authors during the research.

These are just a few examples of the algorithms used in cloud computing for VM placement. Different cloud providers may use different algorithms based on their specific requirements and goals.

According to the typical behavior of a cloud system, when a new request is received, the MRM and GM attempt to locate where the new machine will be started. This process follows the physical resource-allocation algorithm implemented in the system, examples of which are described above. For the study, the authors assumed an even distribution between all available physical machines in the system. It was also assumed that the activation of a new VM on a PM can only occur if a single PM has sufficient free resources defined by a call consisting of the necessary amount of RAM, the necessary disk space, the number of CPU cores and, the bit rate to connect to the server.

### 2.2. Physical Machine

As presented by the authors in [27,28], a single physical machine on which VMs are created can be described by four basic parameters:

- $C_{P}$—the number of processors (cores),
- $C_{R}$—the total capacity of RAM,
- $C_{D}$—the total capacity of the hard disk,
- $C_{B}$—the total bitrate of a network link.

A demand for the creation of a new VM of class *i* can be described by the four-element set $VM_{i}=\left\{c_{i,P},c_{i,R},c_{i,D},c_{i,B}\right\}$, where

- $c_{i,P}$—the number of demanded processors (cores),
- $c_{i,R}$—the demands for capacity of RAM,
- $c_{i,D}$—the demanded capacity of the hard disk,
- $c_{i,B}$—the demanded speed of a network link,

where *i* denotes the class of a VM, understood as a group of machines that require identical values of the parameters of set $VM_{i}$. Typically, it is assumed that the number of classes of VM is equal to *m*.

Consequently, for a single physical machine ($PM=(C_{P},C_{R},C_{D},C_{B})$) to be able to create a new virtual machine with requests, respectively ($VM_{i}=\left(c_{i,P},c_{i,R},c_{i,D},c_{i,B}\right)$), it is necessary that the current free number of resources of each type is sufficient to handle this request. The original concept of such a system was first proposed in the article [26], and a model for determining the blocking probability for such a system was proposed by the authors in [28].

## 3. Model

### 3.1. Basic Analytical Models

Models that can be used to analyze ICT systems include analytical and simulation models. These models can be utilized to analyze various aspects of a system, such as network performance, reliability, availability, and security. In this study, the authors conducted research on resource utilization in an infrastructure of cloud computing using basic analytical models of multi-service ICT systems.

The proposed solution uses three models known from the literature on the subject: the full-availability system (FAS) model [29,30], the limited-availability system (LAS) model [31,32], and the fixed-point (FP) method [33]. Using these three methods in a single model made it possible to estimate resource utilization in a complex system such as cloud computing physical infrastructure. To more easily analyze the analytical solution proposed in the article, the remainder of this section presents the most important information about the FAS, LAS, and FP models used.

### 3.2. Full Availability System

**Figure 2:**
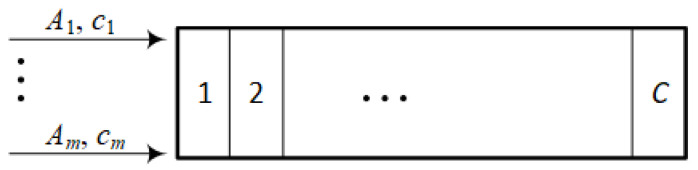
Illustration of the construction of a full availability system.

An ICT system is called a full-availability system if requests occurring at its input have access to any of its resources as long as they are free [29,30]. A simplified schematic of the structure of such a system is shown in Figure 2.

The capacity of the system is *C* units of system capacity, referred to as allocation units (AUs). An AU is an abstract dimensionless unit that allows the capacity of any system to be represented independently of the units in which the actual capacity of the system is expressed. This is extremely important from the point of view of analytical model usage. Modern networks are based on packet transmission. However, network analysis at the packet level is computationally inefficient. In [34], it was shown that ICT systems can be analyzed at the level of packet streams associated with the delivery of the services offered. Such an analysis would not be possible without the process of resource discretization [28,35], which allows the transmission rate to be expressed in dimensionless AUs. Thanks to the discretization of resources, it is possible to also apply the analytical model in cases where the actual capacity of the system is expressed in other units (e.g., in the number of processors or bytes) [26,28]. The system in Figure 2 is offered *m* request classes, each of which requires $c_{i}$ AUs (0≤i≤m) to be serviced. The best-known analytical models of full availability system are the models proposed in [29,30]. The basis of the model is the following recursive equation, which allows us to determine the occupancy distribution in the system:

(1) $$n{\left[P\left(n\right)\right]}_{C}=\sum_{i=1}^{m}A_{i}c_{i}{\left[P\left(n-c_{i}\right)\right]}_{C},$$

where $A_{i}$ is the intensity of traffic of class *i* and ${\left[P\left(n\right)\right]}_{C}$ is the occupancy probability of *n* AUs in FAS with a capacity of *C* AUs.

By knowing the occupancy distribution, it becomes feasible to determine the probability of blocking for each request class serviced by the system under investigation:

(2) $$E_{i}=\sum_{n=C-c_{i}+1}^{C}{\left[P\left(n\right)\right]}_{C}.$$

In a simplified manner, the results of FAS modeling can be symbolically represented as follows:

(3) {P,E}=FAS(A,c,C),

where **P** represents the occupancy distribution obtained based on (1):

(4) $$P=\{{\left[P_{FAS}\left(n\right)\right]}_{C},0⩽n⩽C\},$$

**E** is a set of blocking probabilities obtained based on (2):

(5) $$E=\{E_{i},0⩽i⩽m\},$$

while

(6) $$A=\{A_{1},A_{2},\ldots ,A_{m}\},$$

(7) $$c=\{c_{1},c_{2},\ldots ,c_{m}\},$$

are sets of offered traffic and demands of individual classes of requests.

#### 3.2.1. Limited Availability System

**Figure 3:**
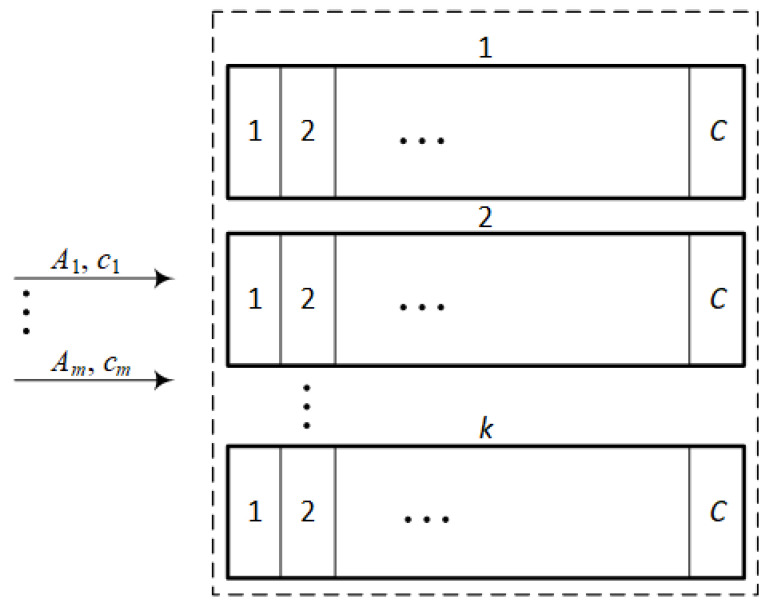
Illustrative drawing of the construction of a limited available system.

Limited availability system (LAS) is a system that is divided into *k* identical, separated FAS subsystems [31]. The capacity of each FAS subsystem is equal to *C* AUs. The system is offered *m* classes of requests, demanding $c_{i}$ AUs (0≤i≤m) for service. An illustrative drawing of a LAS system is shown in the Figure 3.

The system works in such a way that any new request that demands $c_{i}$ AUs can be accepted for service only if it can be completely serviced by a single FAS subsystem. Consequently, it is not possible in this system to divide the request of $c_{i}$ AUs between separate subsystems.

Analytical models for such systems have been proposed in [31,32]. According to these models, the occupancy distribution in LAS can be determined in the following way:

(8) $$n{\left[P\left(n\right)\right]}_{kC}=\sum_{i=1}^{m}kA_{i}c_{i}\sigma_{i}\left(n-c_{i}\right){\left[P\left(n-c_{i}\right)\right]}_{kC},$$

where

- $A_{i}$—the traffic intensity of traffic class *i* offered to a LAS;
- ${\left[P\left(n\right)\right]}_{kC}$—the occupancy probability of *n* AUs in a LAS with a total capacity of kC units, where *C* is the capacity of single subsystem;
- $\sigma_{i}\left(n\right)$—the so-called conditional passage probability for transitions between neighboring occupancy states in a LAS:
  (9) $$\sigma_{i}\left(n\right)=1-\frac{F(kC-n,k,c_{i}-1)}{F(kC-n,k,C)},$$
  where F(x,k,c) is the number of possible distributions of *x* free (unoccupied) AUs in *k* separate resources, where each of the resources has a capacity of *C* units:
  (10) F(x,k,C)=∑i=0xC+1−1ikix+k−1−iC+1k−1.

By knowing the occupancy distribution, it becomes feasible to determine the probability of blocking for each request class serviced by the system under investigation:

(11) $$E_{i}=\sum_{n=0}^{kC}\left[1-\sigma_{i}\left(n\right)\right]{\left[P_{LAS}\left(n\right)\right]}_{kC},$$

In a simplified manner, the results of LAS modeling can be symbolically represented as follows:

(12) {P,E}=LAS(A,c,kC),

where **P** represents the occupancy distribution in LAS, obtained based on (11):

(13) $$P=\{{\left[P_{LAS}\left(n\right)\right]}_{C},0⩽n⩽kC\}.$$

**E** is a set of blocking probabilities (5), obtained based on (2), while **A** and **c** are sets of offered traffic and its demands (formulas (6) and (7)).

#### 3.2.2. Fixed-Point Method

The last model used to develop the algorithm is known in the literature as the fixed-point (FP) method. This method makes it possible to determine the blocking probability in systems in which the new request demands access to several different resources (subsystems) simultaneously. This involves the assumption that to each of the subsystem is offered the traffic (the so-called effective traffic) that is not lost in the other component subsystems of a given system [33]. Such an assumption implies the need to determine the probability according to the adopted algorithm. This algorithm is described in detail by the authors in the article [28] and is as follows:

Request of class *i* (1≤i≤m) demands access to *s* subsystems at the same time. The effective traffic of class *i* requests $A_{i}\left(j\right)$ offered to the subsystem *j* (1≤j≤s) is defined as follows:

(14) $$A_{i}\left(j\right)=A_{i}\prod_{l=1,l\neq j}^{s}\left[1-E_{i}\left(l\right)\right],$$

where $E_{i}\left(l\right)$ is the blocking probability for class *i* requests in the subsystem *j*.

Note that to determine the offered traffic $A_{i}\left(j\right)$, it is necessary to know the value of blocking probability $E_{i}\left(l\right)$ in other subsystems; i.e., all l≠j. Therefore, the FP method is an iterative method that can be implemented in the following way:

1. Initialization of the iteration step: z=0.
2. Determining the initial approximations (z = 0) for the blocking probabilities of all traffic classes in all subsystems:
  (15) $$\underset{i⩽j⩽s}{⋀}E_{j}^{z}=\left\{E_{i,j}^{\left(z\right)},1⩽i⩽m\right\},$$
  where
  (16) $$\underset{i}{⋀}\underset{j}{⋀}E_{i,j}^{\left(0\right)}=0.$$
3. Increasing the iteration step:
  (17) z=z+1.
4. Determining $A_{j}^{\left(z\right)}$, i.e., the effective traffic intensities of individual classes offered to subsystem *j* in step *z*:
  (18) $$\underset{1⩽j⩽s}{⋀}A_{j}^{\left(z\right)}=\left\{A_{1,j}^{\left(z\right)},A_{2,j}^{\left(z\right)},\ldots ,A_{m,j}^{\left(z\right)}\right\},$$
  where each element of set $A_{j}^{\left(z\right)}$ is determined according to the formula:
  (19) $$\underset{1⩽i⩽m}{⋀}\underset{1⩽l⩽s}{⋀}A_{i,j}^{\left(z\right)}=A_{i}\prod_{l=1,l\neq j}^{s}\left[1-E_{i,l}^{\left(z-1\right)}\right].$$
5. Determining $P_{j}^{\left(z\right)}$, $E_{j}^{\left(z\right)}$, i.e., the occupancy distributions and blocking probabilities of individual classes in subsystem *j* at step *z*:
  (20) $$\underset{1⩽j⩽s}{⋀}P_{j}^{\left(z\right)}=\left\{{\left[P_{FAS}\left(n\right)\right]}_{C_{j}}^{\left(z\right)},1⩽n⩽C_{j}\right\},$$
  (21) $$\underset{1⩽j⩽s}{⋀}E_{j}^{\left(z\right)}=\left\{E_{1,j}^{\left(z\right)},E_{2,j}^{\left(z\right)},\ldots ,E_{m,j}^{\left(z\right)}\right\},$$
  where each element of the $P_{j}^{\left(z\right)}$ and $E_{j}^{\left(z\right)}$ sets is determined, respectively, based on (3):
  (22) $$\underset{1⩽j⩽s}{⋀}P_{j}^{\left(z\right)}=FAS\left(A_{j}^{\left(z\right)},c_{j},C_{j}\right),$$
  (23) $$\underset{1⩽j⩽s}{⋀}E_{j}^{\left(z\right)}=FAS\left(A_{j}^{\left(z\right)},c_{j},C_{j}\right),$$
  where $c_{j}$ is a set of requests for individual traffic classes in subsystem *j* with capacity $C_{j}$:
  (24) $$c_{j}=\left\{c_{1,j},c_{2,j},\ldots ,c_{m,j}\right\},$$
6. Determining the total blocking probability $E^{\left(z\right)}$, i.e., the blocking probability values of individual classes in the entire system at step *z*:
  (25) $$E^{\left(z\right)}=\left\{E_{1}^{\left(z\right)},E_{2}^{\left(z\right)},\ldots ,E_{m}^{\left(z\right)}\right\},$$
  where each element of the $E^{\left(z\right)}$ set is determined by the formula:
  (26) $$\underset{1⩽i⩽m}{⋀}\underset{1⩽j⩽s}{⋀}E_{i}^{\left(z\right)}=1-\prod_{j=1}^{s}\left[1-E_{i,l}^{\left(z\right)}\right].$$
7. Checking the accuracy of the calculations:
  (27) $$\underset{i}{⋀}\left|\frac{E_{i}^{\left(z\right)}-E_{i}^{\left(z-1\right)}}{E_{i}^{\left(z\right)}}\right|\leq \epsilon .$$
  If the condition is not met for all *i*, go to Step 3; otherwise, $E^{\left(z\right)}=E$, and the calculations end.

The results of the FP method are symbolically represented as follows:

(28) $$\left\{P_{j},E_{j}\right\}=FP\left(A,c_{j},C_{j}\right),$$

where A is a set of offered traffic defined by (6).

In the presented algorithm, it is assumed that $X^{\left(z\right)}$ is the value of parameter *X* in the *z*-th iteration step. The ϵ parameter is the absolute error of the calculations, which specifies the accuracy of the iteration process.

### 3.3. Proposed Model

The proposed analytical algorithm makes it possible to determine the use of individual resources in physical machines in a cloud system operating in the IaaS model. The algorithm takes into account both the physical architecture of the system and all the basic parameters necessary to create a machine (P,R,D,B), while assuming that the entire virtual machine must be allocated in a single physical machine [28]. At the same time, it was assumed that the entire system consists of *k* identical physical machines, and that the algorithm for allocating VMs to physical machines used by the Cloud Manager seeks to load all machines equally. In summary, a request of class *i* occurring in the system can be fully served if and only if there is at least one physical machine in the system consisting of *k* physical machines in which there is a sufficient number of free AUs necessary to create a new virtual machine. In order to calculate the resource utilization of a cloud system, the Cloud I Algorithm, proposed by the authors in the paper [28], served as the basis. In this algorithm, the LAS and FAS models, as well as the fixed-point method, were used in order to determine the blocking probability in the cloud system. This model, in an initial phase, allows the determination of the distribution considering the individual resources independently for a single machine (FAS) and a group of machines (LAS), then finding the relationship in the obtained loss factors. Each of these models simultaneously allows a distribution of occupancy to be created, which, in subsequent steps, can be used to determine the average use of each resource independently and to determine the relationship coefficients for these parameters as well. At the same time, the fixed-point method makes it possible to determine the occupancy distribution for a single machine, taking into account the simultaneous need for all types of resources to create a virtual machine. In the next step, this translates into the possibility of taking into account the previously determined correlation coefficients of the average utilization of individual, independent resources using LAS and FAS models to determine the utilization in an actual physical machine located in the cloud system. The determined correlation coefficients take into account the presence of multiple physical machines so that the parameters obtained using the fixed-point method can be multiplied by these values.

Let us delve into the idea used in the proposed ARU (Actual Resource Utilization) model. The average resource utilization $L_{FAS}$ in a given FAS system with capacity *C* is determined by the equation

(29) $$L_{FAS}=\sum_{n=0}^{C}n{\left[P_{FAS}\left(n\right)\right]}_{C}.$$

Now, consider a LAS (large aggregation system) consisting of *k* FAS subsystems, such that the traffic intensity offered by LAS and its capacity are k times greater than the traffic offered to the FAS subsystem. In such a system, the utilization of resources $L_{LAS}$ can be defined by the equation:

(30) $$L_{LAS}=\sum_{n=0}^{kC}n{\left[P_{LAS}\left(n\right)\right]}_{kC}.$$

The utilization of resources $L_{LAS}$ in a single LAS subsystem, assuming a uniform distribution of traffic among all subsystems, is determined by the equation

(31) $$l_{LAS}=\frac{L_{LAS}}{k}.$$

Now, let us introduce the resource utilization coefficient ϑ, which defines the ratio of the load on a single LAS subsystem to the load on the FAS system:

(32) $$\vartheta =\frac{l_{LAS}}{L_{FAS}}=\frac{L_{LAS}}{kL_{FAS}}.$$

It is evident that the coefficient ϑ allows us to determine the average resource utilization in LAS based on the average resource utilization in FAS, as well as average resource utilization in FAS based on the average resource utilization in LAS. Such an approach has been employed in the proposed model for determining resource utilization.

Let us consider a system in which we have access to *k* PMs. In such a system, a single PM is regarded as a set of resources:

(33) $$C_{PM}=\left\{C_{P},C_{R},C_{D},C_{B}\right\}.$$

Let us assume that each individual PM is offered requests for VM allocation of class *i*(1⩽i⩽m) in each of the resources belonging to CPM. We will denote these requests in a set as

(34) $$c_{i}=\left\{c_{i,P},c_{i,R},c_{i,D},c_{i,B}\right\}.$$

Now, let us consider the occupancy distributions in individual resources $C_{X}$$\left(C_{X}\in C_{PM}\right)$, assuming at the same time that these resources serve VMs with requests $c_{i,X}$$\left(c_{i,X}\in c_{i}\right)$ independently of handling these requests in the remaining resources $C_{PM}$. Therefore, based on (3) and (4),

(35) $$P_{PM,X}=FAS\left(A_{PM,X},c_{PM,X},C_{X}\right)=\left\{{\left[P_{FAS}\left(n\right)\right]}_{C_{X}},0⩽n⩽C_{X}\right\},$$

where

(36) $$A_{PM,X}=\{\left[A_{i,X},1⩽i⩽m\right\},$$

(37) $$c_{PM,X}=\left\{c_{i,X},c_{i,X}\in c_{i}\wedge 1⩽i⩽m\right\},$$

The distribution $P_{PM,X}$ allows us to determine the average utilization $L_{PM,X}$ of type *X* resources in a single PM

(38) $$L_{PM,X}=\sum_{n=0}^{C_{X}}n{\left[P_{FAS}\left(n\right)\right]}_{C_{X}},$$

Now, let us determine the average utilization of resources $L_{kPM,X}$ in a group of *k* PMs forming an LAS. The system is offered traffic with intensity $A_{kPM,X}$, which is *k* times the multiplied traffic intensity of $A_{PM,X}$:

(39) $$A_{kPM,X}=kA_{PM,X}=\left\{kA_{i,X},1⩽i⩽m\right\},$$

The occupancy distribution in such a LAS, based on (12) and (13), can be expressed as follows:

(40) $$P_{kPM,X}=LAS\left(A_{kPM,X},c_{PM,x},kC_{X}\right)=\left\{{\left[P_{LAS}\left(n\right)\right]}_{kC_{X}},0⩽n⩽kC_{X}\right\},$$

The distribution (40) allows for the direct determination of the average resource utilization $L_{kPM,X}$ in the system composed of *k* PMs:

(41) $$L_{kPM,X}=\sum_{n=0}^{kC_{X}}n{\left[P_{LAS}\left(n\right)\right]}_{kC_{X}},$$

as well as the utilization coefficient ϑ defined in (32). For resources of type *X*, we have

(42) $$\vartheta_{X}=\frac{L_{kPM,X}}{kL_{PM,X}}.$$

In determining $L_{PM,X}$, we assumed that the VM handling in the selected resources $C_{X}\left(X\in P,R,D,B\right)$ is independent of this machine’s request to hand in the remaining PM resources. In reality, VM handling requires the simultaneous allocation of requests $c_{i}$ (Equation (34)) in each resource belonging to the resource set $c_{PM}$ (Equation (33)). Therefore, to determine the occupancy distribution $P_{PM,X}$ in the selected *X*-type resources, the FP method can be used. Thus, according to (28), we can write

(43) $$P_{PM,X}^{*}=FP\left(A_{PM,X},c_{PM,X},C_{X}\right).$$

The obtained occupancy distribution allows for the determination of $L_{PM,X}^{*}$, i.e., the actual average utilization of type *X* resources. According to (38), we can formulate the equation as

(44) $$L_{PM,X}^{*}=\sum_{n=0}^{C_{X}}n\left[P_{FP}^{*}\left(n\right)\right]C_{X},$$

where

(45) $$\left[P_{FP}^{*}\left(n\right)\right]C_{X}\in P_{PM,X}^{*}.$$

Now, utilizing the resource utilization coefficient determined in Equation (42), we can calculate the parameter $L_{kPM,X}^{*}$, which represents the actual average resource utilization of type *X* in a group of *k* PMs. Based on Equation (42), we have

(46) $$L_{kPM,X}^{*}=k\vartheta_{X}L_{PM,X}^{*}$$

The determination of the average resource utilization will be expressed in the form of the ARU method.

#### ARU Method

Summarizing the previous considerations, the ARU method can be represented as follows:

**ARU METHOD:**
  1. Determination, based on (35), of the distributions $P_{PM,X}$ in a single PM for each type of resource (X∈{P,R,D,B}). It is assumed that the resources $C_{X}$ handle VMs with requests $c_{i,X}$ ($c_{i,X}\in c_{i},1⩽i⩽m$), independently of the handling of these VMs in the other resources $C_{PM}$.
  2. Determination—based on (38)—of the average resource utilization $L_{PM,X}$ for each type of PM resource (X∈{P,R,D,B}).
  3. For each type of resource (X∈{P,R,D,B}), determination of the occupancy distributions $P_{kPM,X}$ (formula (40)) in a group of *k* PMs forming LAS. It is assumed that the system offers traffic that is *k* times the multiplicity of the traffic offered by a single PM (Formula (39)).
  4. Determination—based on (42)—of the average resource utilization $L_{kPM,X}$ for each type of resource (X∈{P,R,D,B}) in a group of *k* PMs.
  5. Calculation, for each type of resource *X*, of the resource utilization coefficient $\vartheta_{X}$ (Formula (42)).
  6. Determination, based on (43), of the distributions $P_{PM,X}^{*}$ in a single PM for each type of resource (X∈{P,R,D,B}), assuming that VM handling requires the simultaneous allocation of requests $c_{i}$ (formula (34)) in each resource belonging to the set of resources $C_{PM}$ (Formula (33)).
  7. Determination—based on (44)—of the actual average resource utilization $L_{PM,X}^{*}$ in a single PM for each type of resource *X* (X∈{P,R,D,B}).
  8. Determination of the actual average resource utilization $L_{kPM,X}^{*}$ in a group of *k* PMs for each type of resource *X* (X∈{P,R,D,B}) (Formula (46)).

To determine the actual average resource utilization $L_{kPM,X}^{*}$ in a group of *k* PMs, the resource utilization coefficient $\vartheta_{X}$ was applied, calculated with the assumption that the traffic handling in the specific resources $C_{X}$ of a given PM is independent of the traffic handling in the remaining resources belonging to the set $C_{PM}$. This means that the calculations of the $\vartheta_{X}$ parameter are based on the traffic $A_{PM,X}$ (Equation (36)), directly offered by a single PM. Conversely, the calculations of the actual average resource utilization $L_{PM,X}^{*}$ in a single PM result from the application of the FP method and thus are based on the effective traffic $A_{PM,X}^{\left(z\right)}$ (Equation (18)), determined on the basis of the Equation (18) in the last *z*-iteration of the FP method. Therefore, to determine the actual average resource utilization $L_{kPM,X}^{*}$ in a group of *k* PMs, the resource utilization coefficient $\vartheta_{X}$ (Equation (46)), previously determined for different traffic values, i.e., traffic $A_{PM,X}$, is applied in the ARU method. Simulation studies conducted by the authors have shown that the adopted approximation has little impact on the accuracy of the final results. In the examples analyzed in Section 4, the error introduced by traffic differences does not exceed 1%.

## 4. Results

The proposed calculation method was implemented in the C++ language. This program makes it possible to perform calculations for a given cloud computing infrastructure as a function of offered traffic. Since the proposed method is approximate (i.e., the results obtained are not derived from the solution of a system of linear equations resulting from the analysis of the process of servicing requests in the cloud infrastructure), it is necessary to verify its performance by comparing the obtained results with the results of digital simulation. Therefore, it was necessary to develop and implement a simulator of physical cloud infrastructure. The simulator, implemented in the C++ language, generated systems with “k” identical servers. Each server comprised four fundamental parameters—RAM, hard disk, processors, and Ethernet ports—upon which virtual machines could be created. Employing an event-scheduling method within the C++ simulator, we conducted eight series of simulations, each continuing until 1,000,000 requests from the class demanding the highest number of AUs (the maximum RAM AUs) were processed.

**Figure 4:**
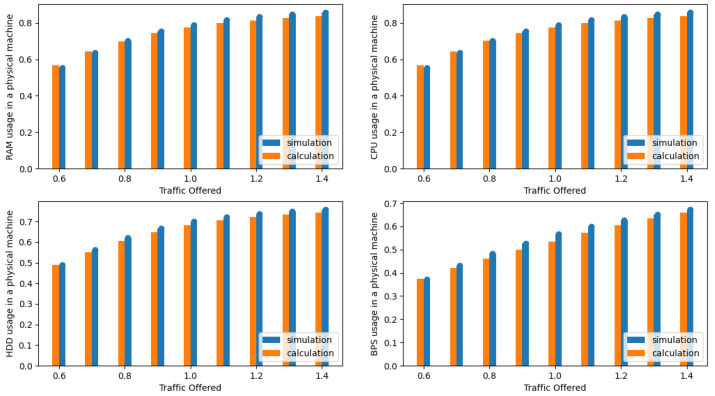
Average resources (R, D, P, B) utilization in a single server for System 0.

**Figure 5:**
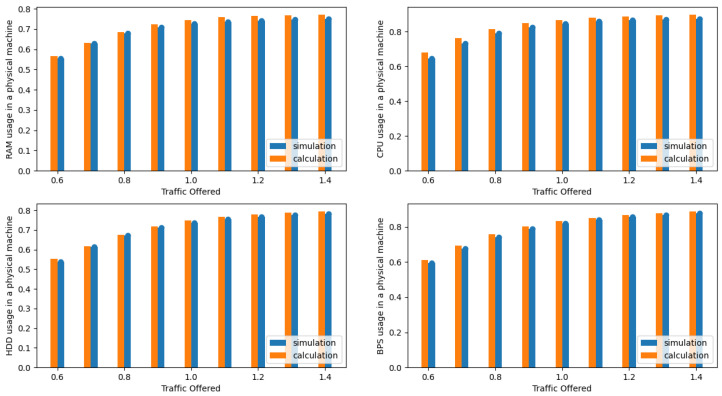
Average resource (R, D, P, B) utilization in a single server for System 1.

**Figure 6:**
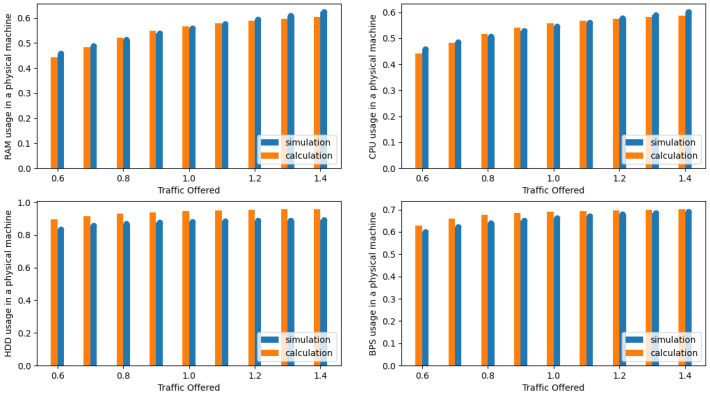
Average resources (R, D, P, B) utilization in a single server for System 2.

**Figure 7:**
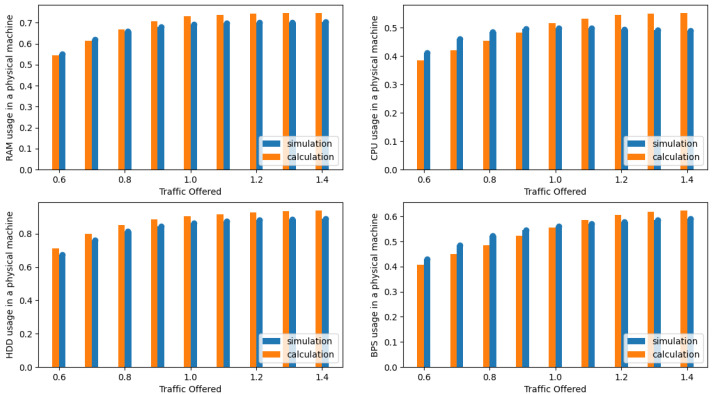
Average resources (R, D, P, B) utilization in a single server for System 3.

**Figure 8:**
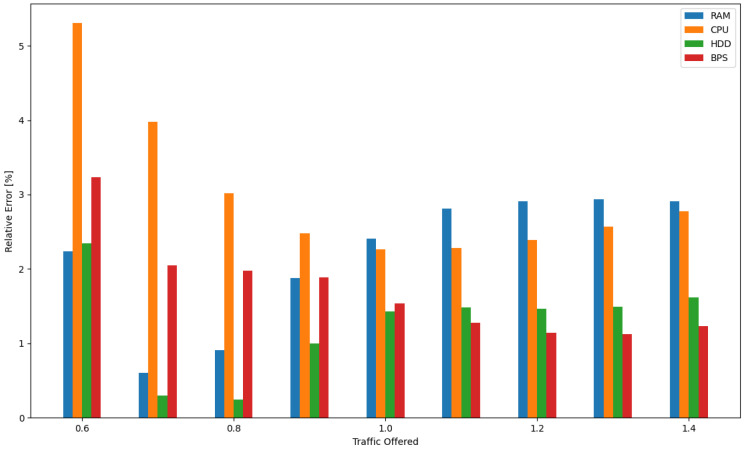
Relative error between the results obtained using the analytical model and the result of simulation studies.

**Figure 9:**
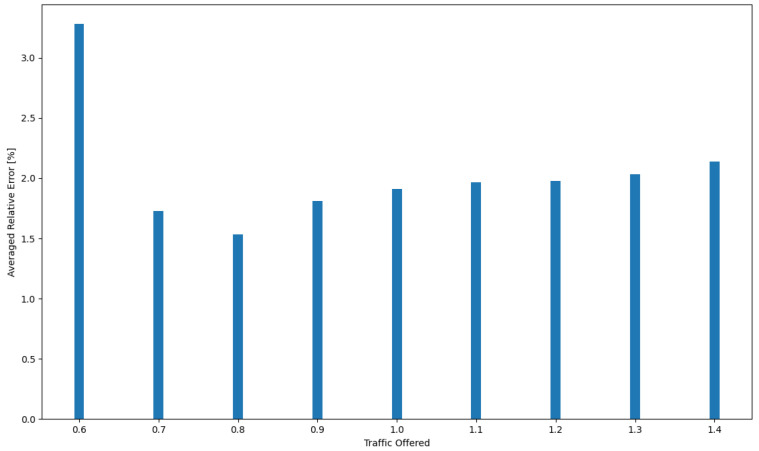
Average relative error for all system resources between the results obtained using the analytical model and the result of simulation studies.

The presented results in the Figure 4, Figure 5, Figure 6, Figure 7, Figure 8 and Figure 9 are shown as a function of show the average traffic offered to one AU:

(47) $$a=\frac{\sum_{i=1}^{m}A_{i}c_{i,R}}{C_{R}}.$$

Traffic offered was divided between the different classes of applications in the following proportions $A_{1}c_{1,R}:A_{2}c_{2,R}:\ldots :A_{m}c_{m,R}$=1:1:…:1.

An essential assumption throughout the system pertained to the conversion of AUs into actual capacity parameters for individual resources. This conversion was determined based on equivalent bandwidth, as outlined in Table 1.

The process of determining the equivalent bandwidth for each system parameter was carried out for the parameters of the virtual machine and physical machine solutions offered on the market. The authors carried out a reconnaissance of the offers available on the market for the purchase of virtual machines available through the Microsoft Azure service.

The research process included five different cases, where System 0 was designed with available Microsoft solutions in mind. The entire System 0 was built with three DELL servers (PowerEdge R7625 model) with the following specifications: 2× AMD EPYCTM 9654 processor with a base clock speed of 2.4 GHz/3.7 GHz. The total number of installed processor cores was 192 (2 × 96), 184 of which were available to users. The server was additionally equipped with 4× 256 GB of DDR4 RDIMM RAM, 736 GB of which were available to users. In addition, a 1.92 TB drive, entirely available to users, was fitted to the server. A 2 × 1 GB network card was also fitted. As mentioned earlier, the system offered virtual machines in line with the machines that are available for purchase under the Microsoft Azure D2s v3–D64s v3 service. The machines selected were D8s v3 (8× CPUs, 16 GBs RAM, and 64 GB disk), D16s v3 (16× CPUs, 32 GBs RAM, and 128 GB disk), and D32s v3 (32× CPUs, 64 GBs RAM, and 256 GB disk). An equal link to all devices was assumed at the 100 Mbps link guarantee level.

Other analyses (System 1–System 4) were performed to demonstrate the independence of the model from the system parameters, the combination of both requests, and the parameters of the physical machines themselves.

### The Use of Individual Physical Machine Resources in the System

As part of the study, the authors conducted a series of tests using the author’s simulator. The tests were carried out on a number of different systems, of which the results for four are described in detail in the article. Throughout the simulation process, the use of cloud system resources over time was analyzed to provide data on their average utilization. Parameters such as RAM, disk, processor (CPU usage), and bandwidth on the server were monitored. These values were then aggregated after a series of simulations and normalized by the total simulation time.

**Figure 10:**
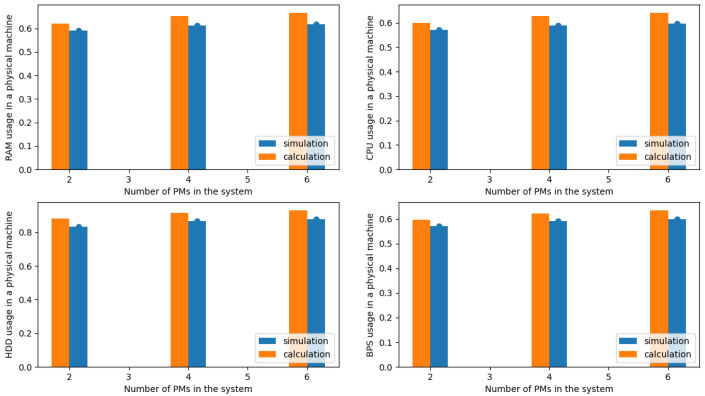
Average resources (R, D, P, B) utilization in a single server for System 4.

In the next phase, the authors compared the results obtained from the simulation with the results obtained with the authors’ proposed analytical model (see Section 3.3). Figure 4, Figure 5, Figure 6 and Figure 7 illustrate the results obtained for Systems 0–3 in which the traffic offered per unit capacity of the whole system varied between 0.6 and 1.4 Erl. Next, Figure 10, illustrates the resource utilization results for individual physical machines, where the traffic offered for the system was constant at 1 Erl (System 4) per unit capacity of the whole system, while the number of physical machines varied between 2 and 6. In all figures, the orange color indicates the results obtained using the analytical model (ARU method), while the blue color indicates the results obtained from simulation studies.

The detailed specifications of System 0–3 are shown in Table 2 and of System 4 are shown in Table 3.

The analytical model developed by the authors showed a high level of accuracy, with a slight tendency to overestimate resource utilization. Due to its high accuracy, it can be a useful tool in the design process of cloud systems based on IaaS architecture. A careful analysis of the maximum relative error between the results obtained with the simulation model and the analytical model confirmed the high accuracy of the proposed solution. The maximum relative error for all parameters monitored in the system (R, D, P, B) for System 1 did not exceed 6%. The consistency of the obtained results for all analyzed parameters and their similar accuracy confirm that the developed solution can be effectively used to design cloud systems even with high QoS requirements.

The resulting independence of offered traffic and incoming request structures further strengthens the reliability of the approach. As a result, the simulation model and analytical algorithm not only demonstrate efficiency in the cloud system design process but also present a high level of accuracy, with the maximum relative error within acceptable limits. Moreover, in order to clearly convey the precision of the analytical model, the authors included a comprehensive visual representation of the relative error in Figure 8. This figure illustrates the maximum relative error between the simulation results and the analytical model. The Table 4 shows the relative errors between simulation results and calculations made using the developed model for RAM, CPU, disk, and bandwidth for System 1, respectively, while Figure 9 shows the averaged relative error obtained for all resources as a function of traffic offered. Thus, if one were to determine the average relative error for System 1 across all resources for each traffic offered and then average these values, the average relative error across the system would be 2.04%.

Conducting experimental tests on a variable spectrum of the number of physical machines varying from 2 to 6 units confirmed the validity of previous observations and served to highlight the robustness of the model. The results for such a system are shown in Figure 10. The findings indicate significant independence of the model in terms of both the number of physical machines deployed and the dynamics of the distribution of requests between different classes, as well as the intensity of incoming requests.

The research provides valuable information on the adaptability and resilience of the model under different operational scenarios. The observed independence from the number of PMs and the distribution of requests between classes indicate that the model can be applied in different deployment scenarios, demonstrating its versatility and generalizability.

## 5. Summary

This paper presents results on the average utilization of individual physical machine resources (RAM, disk, processor (CPU usage), and bandwidth) in an IaaS Cloud environment. These results were obtained based on an approximate analytical model (the ARU method) developed by the authors, which was then compared with digital simulation results to confirm its accuracy. The authors used the event scheduling simulation methodology to create a cloud computing simulation model. The conducted experiments confirmed the accuracy of the developed model, keeping the relative error between the obtained results within an acceptable range even with high QoS requirements. The research conducted provides valuable insight into optimizing cloud systems at the design stage to achieve resource utilization at the desired level. As part of future work, the authors intend to consider a container-based approach within data centers, as well as analysis for different distributions with uniform VMs across physical machines. In addition, the authors intend to consider the possibility of moving VMs between different physical machines, as well as the possibility of scaling the size of VMs at runtime.

## Figures and Tables

**Table 1:** AU definitions.

RAM	1 AU = 16 GB
Processor	1 AU = 8 cores
Disk	1 AU = 64 GB
Bandwidth	1 AU = 100 Mbps

**Table 2:** Parameters of Systems 0–3.

System 0				
Servers				
No. of PMs	Capacity of Server Components in AUs			
k=3	$C_{R}=23$	$C_{P}=23$	$C_{D}=26$	$C_{B}=20$
Virtual Machines				
VM Class	VM Demands in AUs			
1	$c_{1,P}=1$	$c_{1,R}=1$	$c_{1,D}=1$	$c_{1,B}=1$
2	$c_{2,R}=2$	$c_{2,P}=2$	$c_{2,D}=2$	$c_{2,B}=1$
3	$c_{3,R}=4$	$c_{3,P}=4$	$c_{3,D}=4$	$c_{1,B}=1$
System 1				
Servers				
No. of PMs	Capacity of Server Components in AUs			
k=3	$C_{R}=35$	$C_{P}=30$	$C_{D}=28$	$C_{B}=20$
Virtual Machines				
VM Class	VM Demands in AUs			
1	$c_{1,P}=1$	$c_{1,R}=1$	$c_{1,D}=1$	$c_{1,B}=1$
2	$c_{2,R}=2$	$c_{2,P}=3$	$c_{2,D}=2$	$c_{2,B}=1$
3	$c_{3,R}=3$	$c_{3,P}=3$	$c_{3,D}=1$	$c_{1,B}=1$
System 2				
Servers				
No. of PMs	Capacity of Server Components in AUs			
k=3	$C_{R}=38$	$C_{P}=35$	$C_{D}=27$	$C_{B}=25$
Virtual Machines				
VM Class	VM Demands in AUs			
1	$c_{1,R}=1$	$c_{1,P}=1$	$c_{1,D}=3$	$c_{1,B}=1$
2	$c_{2,R}=3$	$c_{2,P}=3$	$c_{2,D}=3$	$c_{2,B}=4$
3	$c_{3,R}=5$	$c_{3,P}=4$	$c_{3,D}=1$	$c_{3,B}=2$
System 3				
Servers				
No. of PMs	Capacity of Server Components in AUs			
k=9	$C_{R}=21$	$C_{P}=25$	$C_{D}=21$	$C_{B}=18$
Virtual Machines				
VM Class	VM Demands in AUs			
1	$c_{1,R}=1$	$c_{1,P}=1$	$c_{1,D}=2$	$c_{1,B}=1$
2	$c_{2,R}=3$	$c_{2,P}=1$	$c_{2,D}=2$	$c_{2,B}=2$
3	$c_{3,R}=3$	$c_{3,P}=4$	$c_{3,D}=3$	$c_{3,B}=1$

**Table 3:** Parameters of System 4.

System 4				
Servers				
Traffic [Erl]	Capacity of Server Components in AUs			
a=1	$C_{R}=25$	$C_{P}=24$	$C_{D}=26$	$C_{B}=20$
Virtual Machines				
VM Class	VM Demands in AU			
1	$c_{1,P}=1$	$c_{1,R}=1$	$c_{1,D}=2$	$c_{1,B}=1$
2	$c_{2,R}=2$	$c_{2,P}=2$	$c_{2,D}=3$	$c_{2,B}=1$
3	$c_{3,R}=4$	$c_{3,P}=3$	$c_{3,D}=3$	$c_{1,B}=3$

**Table 4:** The relative error between the resource utilization values determined using the analytical model and those obtained through simulation for System 1.

Relative Error [%]				
**Traffic Offered**	**RAM**	**CPU**	**HDD**	**B**
0.6	2.23649795	5.302968054	2.348080821	3.23421324
0.7	0.603117214	3.974417961	0.296085737	2.048389148
0.8	0.912760692	3.015055681	0.242153371	1.973825727
0.9	1.875510402	2.48183634	1.001852035	1.890306685
1	2.411807539	2.267414437	1.431078475	1.533685952
1.1	2.813128107	2.281697061	1.482090429	1.278598927
1.2	2.912439866	2.389068886	1.465307318	1.142268669
1.3	2.938155722	2.572646504	1.492920088	1.128719787
1.4	2.909306255	2.777580047	1.622203661	1.236359382

## Data Availability

The data presented in this study are available upon request from the corresponding author. The data are not publicly available due to the project’s limitations.
